# Studying perceptual bias in favor of the from-above Necker cube perspective in a goal-directed behavior

**DOI:** 10.3389/fpsyg.2023.1160605

**Published:** 2023-09-19

**Authors:** Alexander Kuc, Vladimir Maksimenko, Andrey Savosenkov, Nikita Grigorev, Vadim Grubov, Artem Badarin, Victor Kazantsev, Susanna Gordleeva, Alexander Hramov

**Affiliations:** ^1^Baltic Center for Neurotechnology and Artificial Intelligence, Immanuel Kant Baltic Federal University, Kaliningrad, Russia; ^2^Center for Technologies in Robotics and Mechatronics Components, Innopolis University, Innopolis, Russia; ^3^Neurodynamics and Cognitive Technology Laboratory, Lobachevsky State University of Nizhny Novgorod, Nizhny Novgorod, Russia; ^4^Neuroscience Research Institute, Samara State Medical University, Samara, Russia

**Keywords:** ambiguous images, Necker cube, perceptual bias, top-down processing, bottom-up processing, EEG

## Abstract

When viewing a completely ambiguous image, different interpretations can switch involuntarily due to internal top-down processing. In the case of the Necker cube, an entirely ambiguous stimulus, observers often display a bias in perceptual switching between two interpretations based on their perspectives: one with a from-above perspective (FA) and the other with a from-below perspective (FB). Typically, observers exhibit a priori top-down bias in favor of the FA interpretation, which may stem from a statistical tendency in everyday life where we more frequently observe objects from above. However, it remains unclear whether this perceptual bias persists when individuals voluntarily decide on the Necker cube's interpretation in goal-directed behavior, and the impact of ambiguity in this context is not well-understood. In our study, we instructed observers to voluntarily identify the orientation of a Necker cube while manipulating its ambiguity from low (LA) to high (HA). Our investigation aimed to test two hypotheses: (i) whether the perspective (FA or FB) would result in a bias in response time, and (ii) whether this bias would depend on the level of stimulus ambiguity. Additionally, we analyzed electroencephalogram (EEG) signals to identify potential biomarkers that could explain the observed perceptual bias. The behavioral results confirmed a perceptual bias in favor of the from-above perspective, as indicated by shorter response times. However, this bias diminished for stimuli with high ambiguity. For the LA stimuli, the occipital theta-band power consistently exceeded the frontal theta-band power throughout most of the decision-making time. In contrast, for the HA stimuli, the frontal theta-band power started to exceed the occipital theta-band power during the 0.3-s period preceding the decision. We propose that occipital theta-band power reflects evidence accumulation, while frontal theta-band power reflects its evaluation and decision-making processes. For the FB perspective, occipital theta-band power exhibited higher values and dominated over a longer duration, leading to an overall increase in response time. These results suggest that more information and more time are needed to encode stimuli with a FB perspective, as this template is less common for the observers compared to the template for a cube with a FA perspective.

## 1. Introduction

Processing visual information is an essential function of the brain that helps us interact with the environment. Neuroscientists agree that visual processing involves a combination of bottom-up and top-down components. The bottom-up component is driven by sensory processes that direct our attention to salient features of stimuli and process sensory information in visual areas. Invasive recordings have provided evidence that visual processing follows a bottom-up progression, from V1 to V4 (Melloni et al., 2012; Richter et al., 2017). Each subsequent processing stage requires more sensory information and leads to a more comprehensive and unambiguous interpretation of the stimulus. Thus, at lower stages, we encode individual elements of the stimulus, while at later stages, we interpret the stimulus as a whole object.

In contrast, the top-down component relies on internal processes and utilizes information stored in our memory. The interaction between these two components suggests that the brain compares interpretations encoded in the visual cortex with templates stored in working memory at every stage of visual processing. During earlier stages, only a limited amount of salient information is encoded in the visual cortex, and its correspondence with the template is minimal. As the amount of visual information increases and the stimulus representation becomes clearer in subsequent stages, it is more likely to match the template. This optimization of processing allows observers to make decisions using limited sensory information.

The role of the top-down component becomes more prominent when observers encounter ambiguous sensory information (Fan et al., 2020). On one hand, the top-down component enables the use of contextual cues and past experiences to resolve ambiguity. On the other hand, subjective decisions and the risk of errors may increase. Therefore, it is crucial to better understand the interaction between top-down and bottom-up components in ambiguous processing in order to predict and minimize errors.

When subjects observe a completely ambiguous image, its different interpretations involuntarily switch due to the influence of the internal top-down processing component. This has been reported for various ambiguous images, such as the Rubin vase (Parkkonen et al., 2008) and the Necker cube (Kornmeier et al., 2017b).

An intriguing observation is that subjects may exhibit a bias toward a particular interpretation of ambiguous stimuli. For example, when viewing the ambiguous Necker cube, observers demonstrate a bias in perceptual switching between two interpretations that may arise from the differences in their perspectives: one interpretation has a “from-above” perspective (FA), while the other has a “from-below” perspective (FB) (Kornmeier et al., 2017b). Typically, observers display a priori top-down bias in favor of the FA interpretation, which could reflect a common statistical tendency of looking down more frequently than up at objects. In contrast, individuals with autism spectrum disorder (ASD) do not show a FA bias. This could be explained by the fact that ASD patients rely more on small sensory details and struggle with integrating spatial context and previous perceptual experiences. These results suggest that perceptual bias arises from top-down processes that facilitate our perception when sensory information is ambiguous, while ASD patients predominantly rely on sensory-driven (bottom-up) processes, resulting in the absence of perceptual bias.

Limited knowledge exists regarding perceptual bias in goal-directed behavior when subjects voluntarily choose the interpretation of the Necker cube. In our recent study, we instructed participants to distinguish between left-oriented and right-oriented Necker cubes and observed faster responses to the left-oriented stimuli. This finding confirmed previous research as left-oriented cubes corresponded to the FA perspective. Interestingly, as the ambiguity of the stimulus increased, response times hardly differed between the interpretations (Maksimenko et al., 2021). These findings indicate that observers may alter their processing strategy when information becomes more ambiguous, but further analysis is needed to understand the roles of top-down and bottom-up processing components in these strategies. Furthermore, we used a traditional depiction of the Necker cube where the left-oriented stimuli had a FA perspective and the right-oriented stimuli had a FB perspective. Consequently, it remained unclear whether the observed response time bias was due to the orientation or perspective of the stimuli.

In this study, we expanded upon our previous experimental paradigm by presenting a traditional depiction of the Necker cube and its mirrored projection, which allowed us to examine both left-oriented and right-oriented stimuli from both a “from-above” and a “from-below” perspective. We collected behavioral responses and tested two hypotheses: (i) whether the perspective (FA vs. FB) influences response time, and (ii) whether the effect of perspective on response time depends on the level of stimulus ambiguity. Additionally, we conducted EEG analysis to identify biomarkers that can elucidate different processing mechanisms and explain perceptual bias. In summary, our behavioral results confirmed a shorter response time for the FA perspective and its disappearance under conditions of high ambiguity. The EEG analysis revealed differences in spectral power between the processing of Necker cubes with high ambiguity (HA) and low ambiguity (LA). Based on these observed differences, we proposed potential processing strategies for HA and LA stimuli and explained the response time bias in the goal-directed behavior.

## 2. Materials and methods

**Figure 1:**
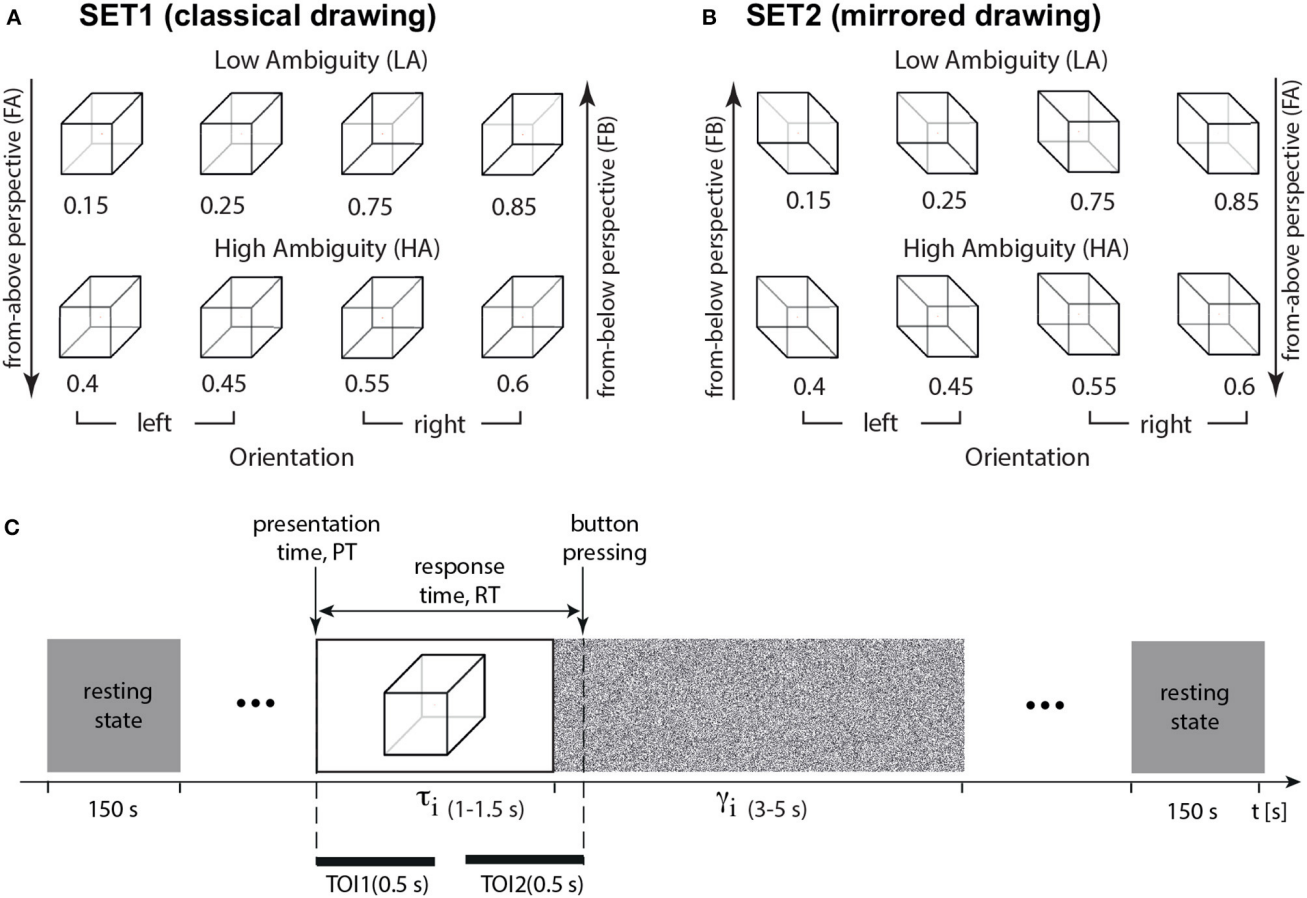
Experimental paradigm. **(A)** The set of visual stimuli, Necker cubes (SET1, classical drawing), with different ambiguity *a* including high-ambiguity (HA), low-ambiguity (LA), left-oriented, and right-oriented Necker cubes (left-oriented stimuli have a from-above perspective, FA, and right-oriented stimuli—from-below perspective, FB); **(B)** the set of visual stimuli, Necker cubes (SET2, mirrored), with different ambiguity *a* including high-ambiguity (HA), low-ambiguity (LA), left-oriented, and right-oriented Necker cubes (left-oriented stimuli have a FB perspective, and right-oriented stimuli – FA perspective); **(C)** structure of the experimental session, τ_*i*_ is the duration of the *i*-th cube presentation, γ_*i*_ is the interval between the *i*-th and (*i* + 1)-th presentations, RT is a response time, TOI1 and TOI2 stand for the time-regions of interest: TOI1—percept-related interval (0.5 s post-stimulus onset), and TOI2—decision-related interval (0.5 s before button pressing).

We conducted two experiments. Experiment 1 included a classical drawing of the Necker cube images (SET1, Figure 1A). We measured behavioral performance (response time and correctness) and neural activity (EEG signals). Experiment 2 included a classical drawing of the Necker cube images (SET1) and its mirrored projection (SET2, Figure 1B). We measured behavioral performance (response time and correctness) without EEG recording.

### 2.1. Experiment 1

#### 2.1.1. Participants

Sixty-one healthy subjects (30 females) aged 18–33 years (20.2 ± 2.5 SD) with normal or corrected-to-normal visual acuity participated in the experiment. All of them provided written informed consent in advance. All participants were naïve. The study was approved by the local ethics committee of the Lobachevsky State University of Nizhny Novgorod (ethical approval number 2, dated 19 March 2021) and was following the Declaration of Helsinki, except for registration in a database.

#### 2.1.2. Stimuli

We chose an ambiguous 2D drawing of a Necker cube as a bistable visual stimulus (Wang et al., 2013; Kornmeier et al., 2017a; Maksimenko et al., 2019, 2020). A subject without perceptual abnormalities interprets this 2D image as a left- or right-oriented 3D object. The ambiguity and orientation of the 3D cube depend on the balance between the brightness of the inner edges forming a left-lower (*b*_*l*_ = 1−*a*) and right-upper (*b*_*r*_ = *a*) squares on the 2D image, *a*∈[0, 1] was a normalized edge's luminance in a gray-scale palette. Thus, the limit cases of *a* = 0 and *a* = 1 corresponded to unambiguous 2D projections of left- and right-oriented cubes, respectively, whereas *a* = 0.5 determined a completely ambiguous spatial orientation of the 3D cube. In our experiment, we used a set of the Necker cube images with *a* = {0.15, 0.25, 0.4, 0.45, 0.55, 0.6, 0.75, 0.85} (Figure 1A). On the one hand, this set could be separated into subsets of left-oriented *a* = {0.15, 0.25, 0.4, 0.45} and right-oriented cubes *a* = {0.55, 0.6, 0.75, 0.85}. On the other hand, in accordance with our previous study (Maksimenko et al., 2020), this set could be also divided into low-ambiguous (LA) images *a* = {0.15, 0.25, 0.75, 0.85}, which are easily interpreted by an observer, and high-ambiguous (HA) images *a* = {0.40, 0.45, 0.55, 0.60}, whose interpretations requires more effort. Unlike the other works on ambiguous stimuli processing (Kornmeier and Bach, 2006; Yokota et al., 2014), we did not present completely ambiguous stimuli, such as a fully symmetrical cube with *a* = 0.5. Moreover, we instructed subjects to be as correct as possible. Therefore, we supposed that the subjects responded on the cube orientation based on the acquired sensory information, rather than the internal representations (Engel and Fries, 2010).

#### 2.1.3. Procedure

During the experiment, participants were comfortably seated in a reclining chair. They held a two-button input device connected to the amplifier with both hands. Participants were instructed to remain relaxed with their eyes open throughout the entire experiment unless instructed otherwise for a specific task. EEG signals were recorded continuously during the entire experiment, including a 3-min rest-state recording period both before and after the task. The Necker cube images, measuring 25.6 cm, were presented on a 27-inch LCD screen with a resolution of 1,920 × 1,080 pixels and a refresh rate of 60 Hz. The screen was positioned at a distance of 2 meters from the participant.

The timing of Necker cubes presentations and the EEG streams were synchronized using a photodiode connected to the amplifier. During experimental sessions, the cubes with predefined ambiguity (SET1 in Figure 1) were randomly demonstrated 400 times, and each cube with a particular ambiguity was presented about 50 times. The experiment lasted around 45 min.

We randomized parameter *a* in the following way. First, we formed a vector *A*(1…400), including all images (50 images for each value of *a*). Then, we randomized indexes in this vector by using the function *randperm* in MATLAB. It returned a row vector containing a random permutation of the indexes from 1 to 400 without repeating elements. Finally, this randomized vector of indexes determined the order of stimuli presentation. We randomized the time of the stimuli presentations and pauses between them as *t*_*min*_ + rand*(*t*_*max*_ − *t*_*min*_). Here, *t*_*max*_ and *t*_*min*_ defined minimal and maximal presentation/pause time, and *rand* is a MATLAB function that returns a single uniformly distributed random number in the interval (0, 1). Each *i*-th stimulus exhibition lasted for a time interval of τ and varied from τ_*min*_ = 1 s to τ_*max*_ = 1.5 s. Pauses, γ between the subsequent presentations of the Necker cube images contained the abstract picture exhibition and varied from γ_*min*_ = 3 s to γ_*max*_ = 5 s (Figure 1D). We instructed participants to press either the left or right key, responding to the left or the right stimulus orientation. We estimated a behavioral response for each stimulus by measuring the response time, RT, which corresponded to the time passed from the stimulus presentation to button pressing (Figure 1D). For each participant, we calculated error rate (ER) as the percentage of erroneous responses. The correctness of each response was evaluated by comparing the actual stimulus orientation with the subject's response. The actual orientation of the Necker cube was defined by the contrast of the inner edges. Thus, a = 0.15, 0.25, 0.4, 0.45 defined the left-oriented cubes, while a = 0.55, 0.6, 0.75, 0.85 stood for the right-oriented ones. To define the correctness, we checked whether the subject pressed the left button for a = 0.15, 0.25, 0.4, 0.45, or the right button for a = 0.55, 0.6, 0.75, 0.85. Otherwise, their response was incorrect.

#### 2.1.4. EEG recording

We registered electroencephalograms (EEG) using a 48-channel NVX-52 amplifier (MKS, Zelenograd, Russia). EEG signals were recorded from 32 standard Ag/AgCl electrodes (Fp1, Fp2, F3, Fz, F4, Fc1, Fc2, F7, Ft9, Fc5, F8, Fc6, Fc10, T7, Tp9, T8, C3, Cz, C4, Cp5, Cp1, Cp2, Cp6, Cp10, P7, P3, Pz, P4, P8, O1, Oz, O2), placed according to the international 10-10 system. The earlobe electrodes were used as a reference. The ground electrode was placed on the forehead. Impedance was kept below 10 KΩ. EEG was digitized with a sampling rate of 1,000 Hz. A band-pass FIR filter filtered the raw EEG signals with cut-off points at 1 Hz (HP) and 100 Hz (LP) and with a 50-Hz notch filter by embedded a hardware-software data acquisition complex. Eyes blinking and heartbeat artifacts were removed by Independent Component Analysis using EEGLAB software (Delorme and Makeig, 2004).

### 2.2. Experiment 2

#### 2.2.1. Participants

Twenty naive healthy subjects (10 females) aged 18–26 years (M = 19.8, SD = 2.4) with no previous psychiatric or neurological history participated in the experiments. All subjects have normal or corrected-to-normal visual acuity. Similarly to Experiment 1, They provided written informed consent in advance. The study was approved by the local ethics committee of the Lobachevsky State University of Nizhny Novgorod and was following the Declaration of Helsinki, except for registration in a database.

#### 2.2.2. Stimuli

We used Necker cubes with the inner edges contrast parameter *a* = {0.15, 0.25, 0.4, 0.45, 0.55, 0.6, 0.75, 0.85}. All stimuli were mirrored around the horizontal axis. The task consisted of 16 stimuli (SET1 and SET2 in Figure 1): eight cubes with the different contrast of the inner edges, presented with two possible orientations (0 and 180deg of rotation).

#### 2.2.3. Procedure

Experiment 2 followed the same protocol as Experiment 1. During experimental sessions, the cubes from SET 1 and SET 2 were randomly demonstrated 400 times, each cube with a particular ambiguity, orientation, and projection was presented about 25 times. Participants were instructed to press either the left or right key when recognizing the left or the right stimulus orientation. The experiment lasted around 45 min. For each stimulus, we determined the response time and correctness in a way similar to the *Experiment 1*.

### 2.3. Behavioral data analysis

Statistical analysis of behavioral data followed a methodology, similar to our recent works (Maksimenko et al., 2020, 2021). For Experiment 1, we performed the group-level statistics for the median RT with two within-subject factors: ambiguity and orientation. For the Experiment 2, we performed the group-level statistics for the median RT with three within-subject factors: ambiguity, orientation, and SET. The main effects were evaluated via repeated-measures ANOVA. The *post-hoc* analysis used either paired samples *t*-test or Wilcoxon signed-rank test, depending on the samples' normality. Normality was tested via the Shapiro-Wilk test. We performed a statistical analysis using SPSS.

### 2.4. EEG analysis

We registered EEG data in Experiment 1 for 61 participants. Examining ER, we excluded three participants whose ER lies above the 95th percentile from the consideration. For the rest 57 participants the ER varied from 0.5 to 30.7% (M = 10.46%, SD = 7.27%). Thus, the response accuracy was above chance ensuring that participants based their decisions on the sensory information. For further confidence, we excluded all stimuli with erroneous responses. Similarly to Maksimenko et al. (2021), we divided EEG signals into epochs. For each stimulus, we introduced two epochs. The first (percept-related) epoch had a length of 4 s, and its middle point was time-locked to the stimulus onset. The second (decision-related) epoch had a length of 4 s, but its middle point was time-locked to the button pressing. We calculated wavelet power (WP) in the frequency band of 1 − 40 Hz using the Morlet wavelet for each epoch. The number of cycles, *n* depended on the signal frequency, *f*, as *n* = *f*. We analyzed WP in two time intervals of interest (TOI): TOI1 was a 0.5 c interval following the stimulus presentation; TOI2 was a 0.5 s interval preceding the button pressing. The WP on these intervals was normalized using 1.5 s. prestimulus interval, so that we introduce event-related spectral perturbations (ERSP): ERSP = (WP − WP_*prestim*_)/WP_*prestim*_. All calculations were performed using the Fieldtrip toolbox in MATLAB (Oostenveld et al., 2011). For TOI1 and TOI2 we averaged NWP over time and contrasted (channel-frequency) pairs between the conditions. To contrast the sensor-level ERSP, we used paired *t*-test in conjunction with the non-parametric cluster-based correction for the multiple comparisons and the Monte Carlo randomization. A cluster was significant when the *p*-value was below 0.025, corresponding to a false alarm rate of 0.05 in a two-tailed test. The number of permutations was 2000.

## 3. Results

### 3.1. Response times in the Experiment 1

**Figure 2:**
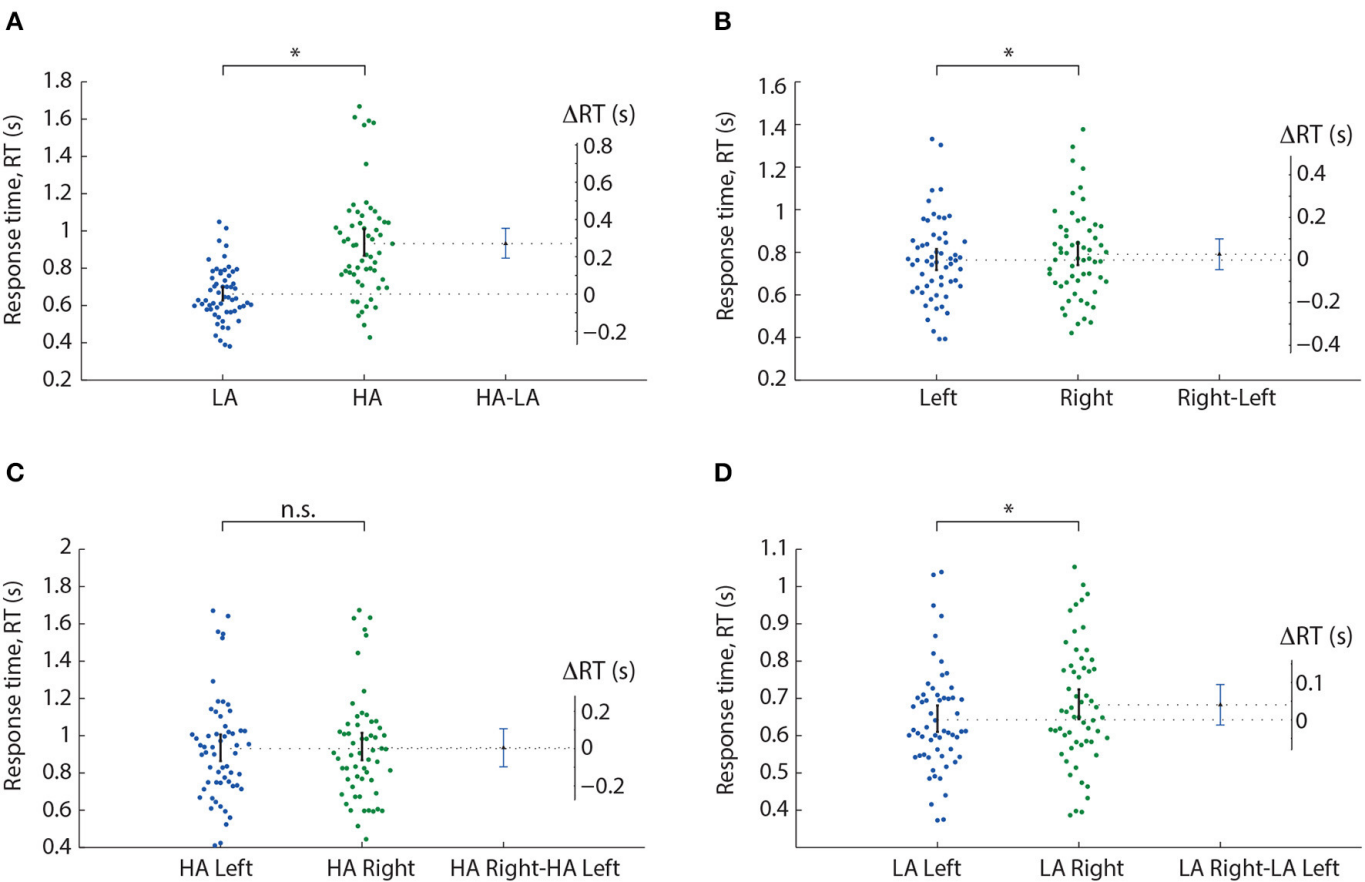
Response time analysis in the Experiment 1. A result of the *post-hoc* comparisons of the median RT between HA and LA stimuli **(A)**, left- and right-oriented stimuli **(B)**, left- and right-oriented HA stimuli **(C)**, and left- and right-oriented LA stimuli **(D)**. Scatter-plot show the median RTs of all subjects, error-bar demonstrates a 95% confidence interval (CI). Difference between groups is shown by the mean value and 95% CI. ^*^p < 0.05 (uncorrected) via a repeated measures ANOVA and the *post-hoc* *t*-test. n.s., not significant.

We analyzed the median RT using a repeated-measures ANOVA with two within-subject factors: ambiguity and orientation (Table 1). As a result, we observed significant main effects of the ambiguity, orientation, and a significant interaction effect, ambiguity * orientation. Thus, we concluded that subjects responded to the left- and right-oriented stimuli differently depending on the ambiguity. The *post-hoc* analysis revealed that the subjects responded faster to the LA stimuli (M = 0.66 s, SD = 0.14) than to HA ones (M = 0.93 s, SD = 0.28): *t*(57) = 11.151, *p* < 0.001 (uncorrected) (Figure 2A). They also responded faster to the left-oriented (M = 0.76 s, SD = 0.19) than to the right-oriented (M = 0.79 s, SD = 0.2) stimuli: *t*(57) = 2.962, *p* = 0.004 (uncorrected) (Figure 2B). When the ambiguity was high, RT to the left-oriented (M = 0.931 s, SD = 0.28 s) and right-oriented stimuli (M = 0.935 s, SD = 0.28 s) was similar: *t*(57) = 0.379, *p* = 0.706 (uncorrected) (Figure 2C). In contrast, for low ambiguity, subjects responded faster to the left-oriented stimuli (M = 0.64 s, SD = 0.14 s) than to the right-oriented ones (M = 0.68 s, SD = 0.16 s): *t*(57) = 4.246, *p* < 0.001 (uncorrected) (Figure 2D).

**Table 1:** The main effects of the ambiguity, orientation, and their interaction on the median response time in the Experiment 1 (ANOVA summary).

**Factors**	***dF*_1_**	***dF*_2_**	**Mean square**	***F***	***p***
Ambiguity (low vs. high)	1	57	4.244	132.002	< 0.001^*^
Orientation (left vs. right)	1	57	0.029	7.643	0.008^*^
Ambiguity * orientation	1	57	0.019	7.235	0.009^*^

^*^significant with *p* < 0.05.

According to our previous work (Maksimenko et al., 2019), the repeated presentation of Necker cube images may induce a training effect resulting in a reduction in response time (RT). Another study (Maksimenko et al., 2021) suggests that RT to the Necker cube image depends on the previously perceived stimulus, with RT to the right-oriented low-ambiguity (LA) stimulus decreasing if the previous stimulus has the same orientation. To ensure that these effects did not influence the RT in our study, we randomized the stimuli following a procedure similar to the ones described in Maksimenko et al. (2019, 2021). We have previously demonstrated that this randomization procedure does not result in significant differences in the median presentation times between conditions. Additionally, the number of previously presented left-oriented and right-oriented stimuli remained the same across conditions.

### 3.2. EEG wavelet power in the Experiment 1

We contrasted ERSP in the TOI1 (percept-related interval) and TOI2 (decision-related interval) in the following cases: (i) between HA and LA cubes; (ii) between the right- and left-oriented cubes; (iii) between the right- and left-oriented HA cubes; (iv) between the right- and left-oriented LA cubes.

#### 3.2.1. HA—LA contrast

**Figure 3:**
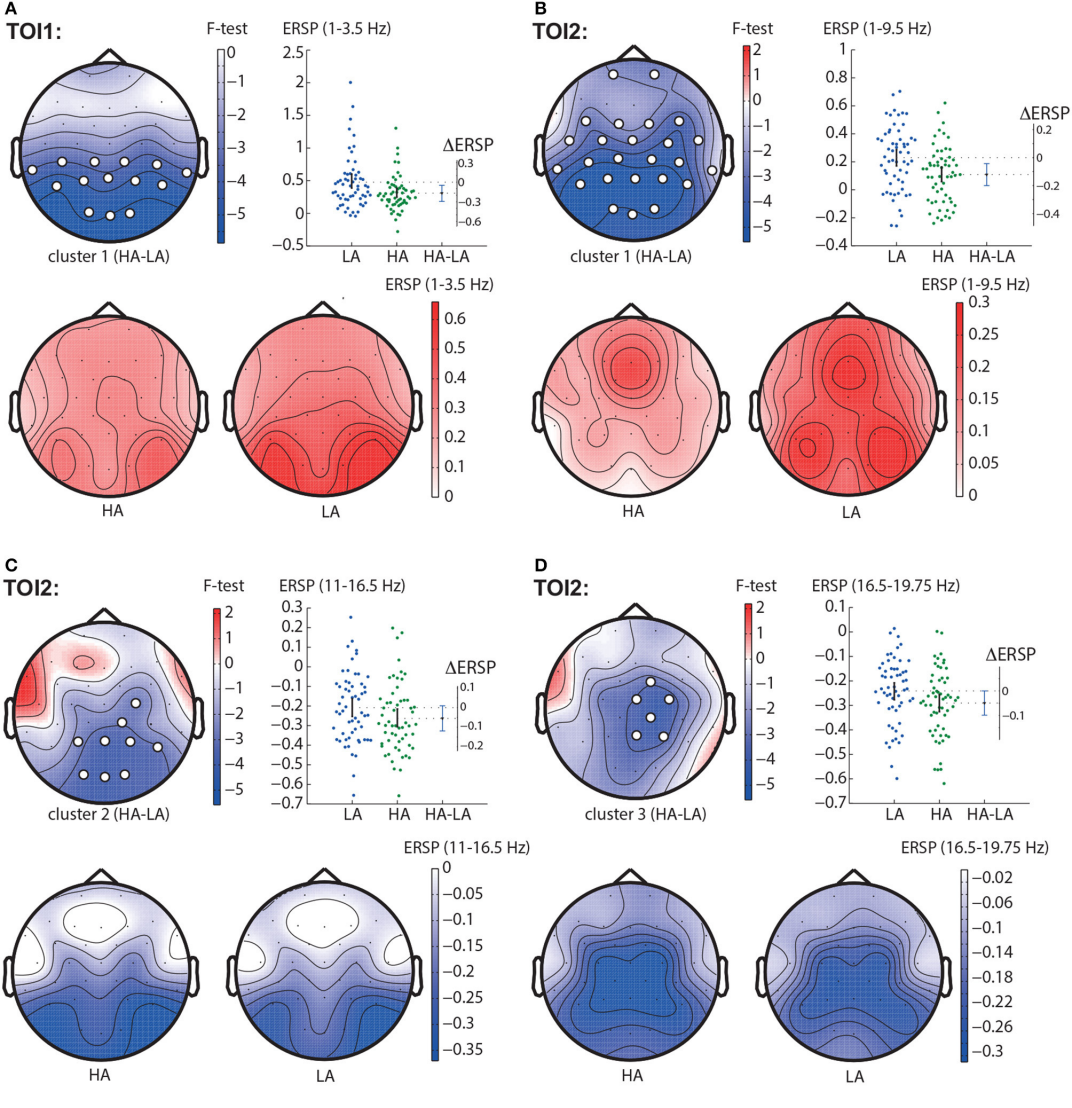
HA-LA contrast. The subfigures illustrate different clusters as the result of ERSP comparison between HA and LA stimuli: the first cluster in the TOI1 **(A)**; the first **(B)**, second **(C)**, and the third **(D)** clusters in TOI2. Topograms reflect the values of *F*-statistic, and the subjects-average ERSP in the compared conditions. Scatter-plot shows the ERSP averaged across the EEG sensors belonging to this cluster. Difference between the conditions is shown with the 95% confidence interval.

In TOI1, the permutation test revealed one negative cluster with *p* < 0.001 in the frequency band of 1 − 3.5 Hz (Figure 3A). This cluster included parietal and central EEG sensors (TP9, CP5, CP1, CP2, CP6, TP10, P7, P3, Pz, P4, P8, O1, Oz, O2). In TOI2, we observed three negative clusters. The first cluster with *p* < 0.001 reflected change in the spectral power in the 1 − 9.5 Hz-band. It included almost all EEG sensors but demonstrated the highest *F*-value in the occipital and parietal areas (Figure 3B). The second cluster with *p* < 0.001 included EEG sensors C4, CP2, CP6, P3, Pz, P4, P8, O1, Oz, O2, and reflected changes in spectral power in the 11.5 − 16.5 Hz frequency band (Figure 3C), The third cluster with *p* = 0.002 appeared in the frequency band of 16.5 − 19.75 Hz, and included EEG sensors FC2, Cz, C4, CP2, Pz, P4 (Figure 3D).

#### 3.2.2. Right—left contrast

**Figure 4:**
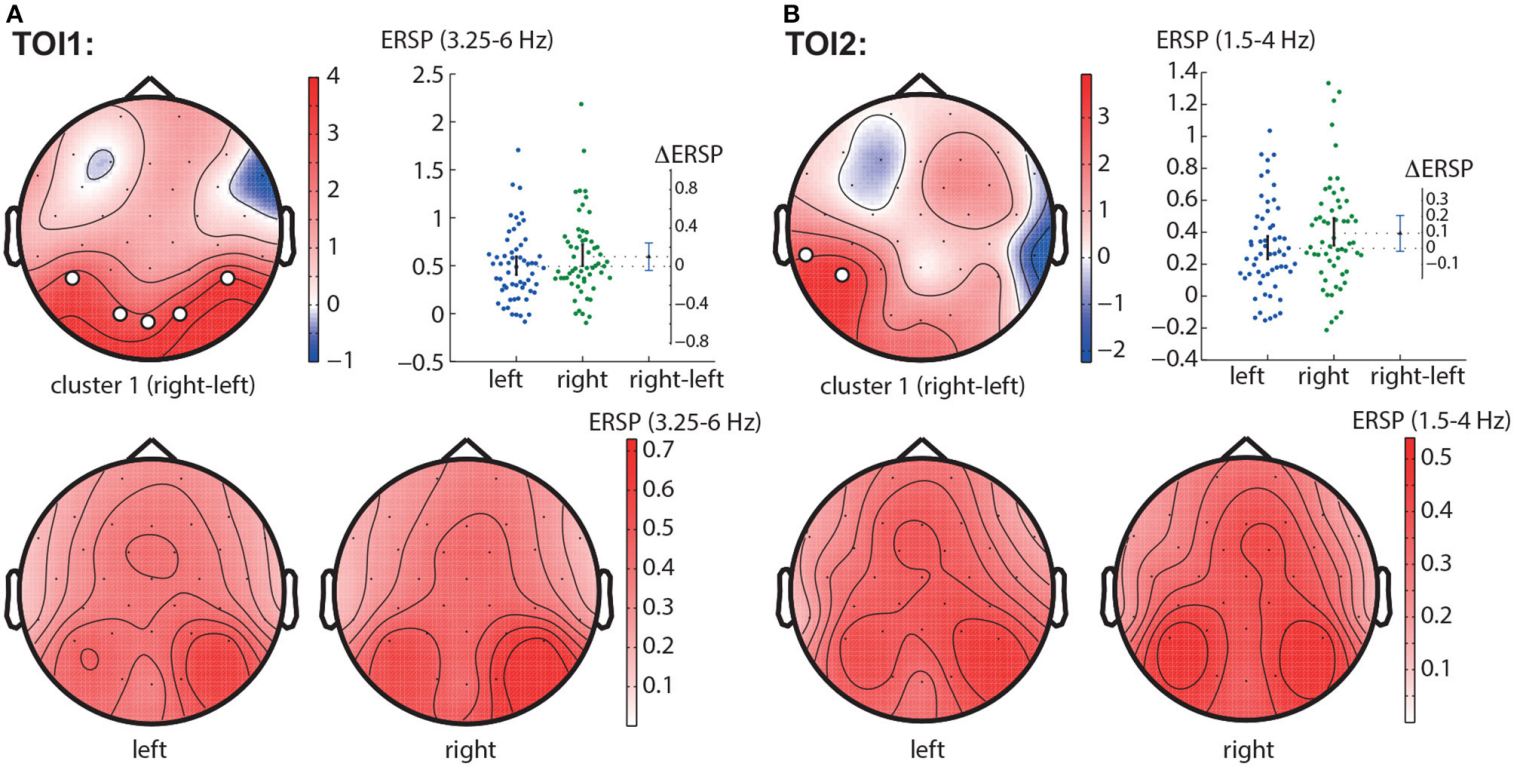
Right-left contrast. The subfigures illustrate different clusters as the result of NWP comparison between the right- and left-oriented stimuli: the first cluster in the TOI1 **(A)** and the first cluster in TOI2 **(B)**. Topograms reflect the values of *F*-statistic, and the subjects-average ERSP in the compared conditions. Scatter-plot shows the NWP averaged across the EEG sensors belonging to this cluster. Difference between the conditions is shown with the 95% confidence interval.

In the TOI1, we observed a negative cluster with *p* = 0.0026 in the frequency band of 3.25 − 6.0 Hz, that included occipito-parietal EEG sensors (P7, P8, O1, Oz, O2) (Figure 4A). In the TOI2, there was a single negative cluster with *p* = 0.0064 in the frequency range of 1.5 − 4 Hz, including EEG sensors TP9, P7 (Figure 4B).

#### 3.2.3. LA right—LA left contrast

**Figure 5:**
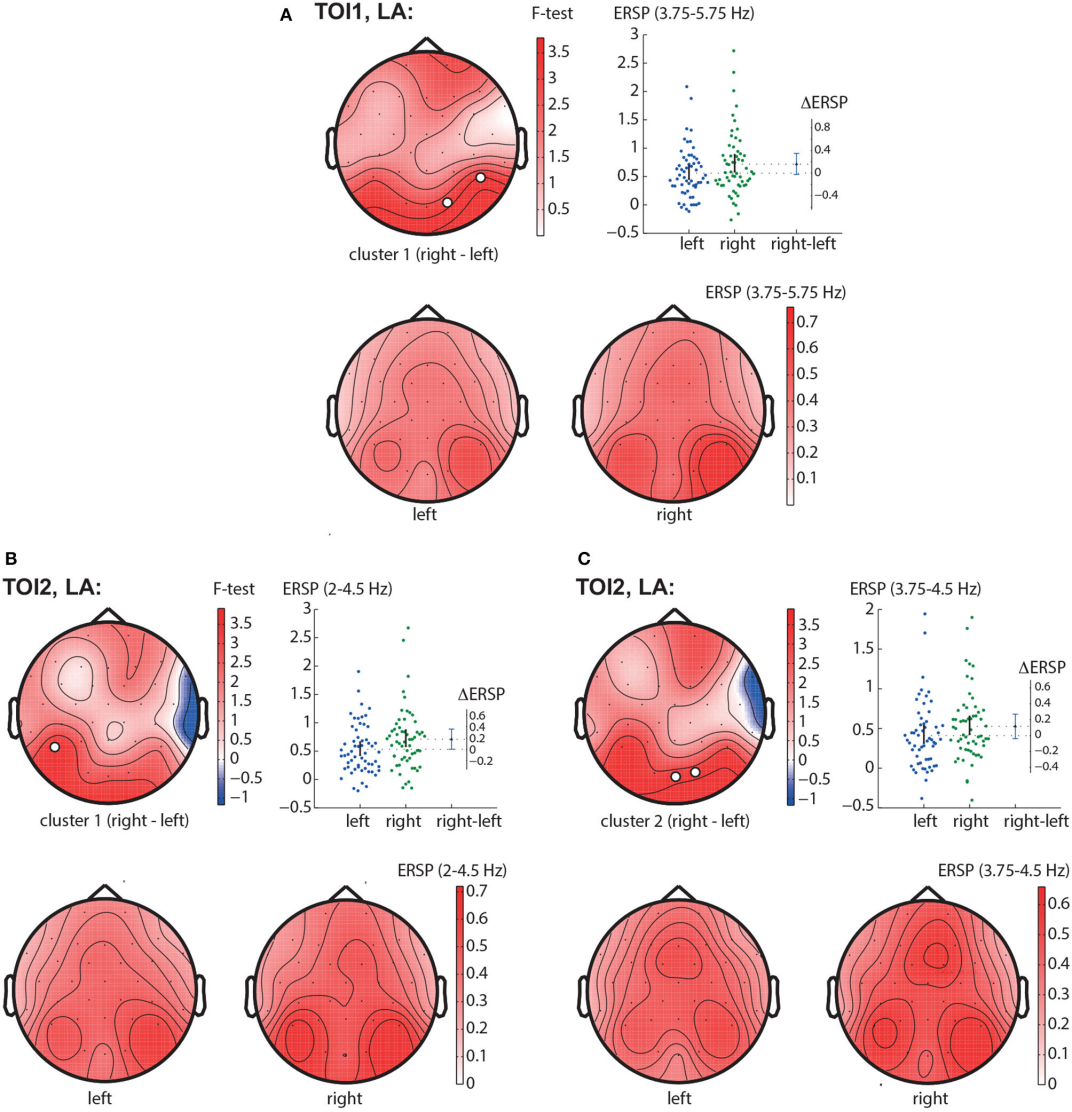
LA right—LA left contrast. The subfigures illustrate different clusters as the result of NWP comparison between the right-oriented and left-oriented LA stimuli: the first cluster in the TOI1 **(A)**; the first **(B)**, and the second **(C)** clusters in TOI2. Topograms reflect the values of F-statistic, and the subjects-average ERSP in the compared conditions. Scatter-plot shows the NWP averaged across the EEG sensors belonging to this cluster. Difference between the conditions is shown with the 95% confidence interval.

In the TOI1, we observed one negative cluster with *p* = 0.0126 in the frequency band of 3.75 − 5.75 Hz, including EEG sensors P8, O2 (Figure 5A). In TOI2, there were two negative clusters. The first cluster with *p* = 0.0146 included EEG sensors P7 and reflected changes in the 2 − 4.5 Hz spectral power (Figure 5B). The second cluster with *p* = 0.0318 included occipital EEG sensors (Oz and O2) and reflected changes in the 3.75 − 4.5 Hz spectral power (Figure 5C). Due to the intersection of the frequency bands and the neighborhood of EEG sensors, we suggest considering them as a single occipito-parietal negative cluster in the theta-band.

#### 3.2.4. HA right-HA left contrast

No clusters were found.

### 3.3. Time evolution of the ERSP in the Experiment 1

**Figure 6:**
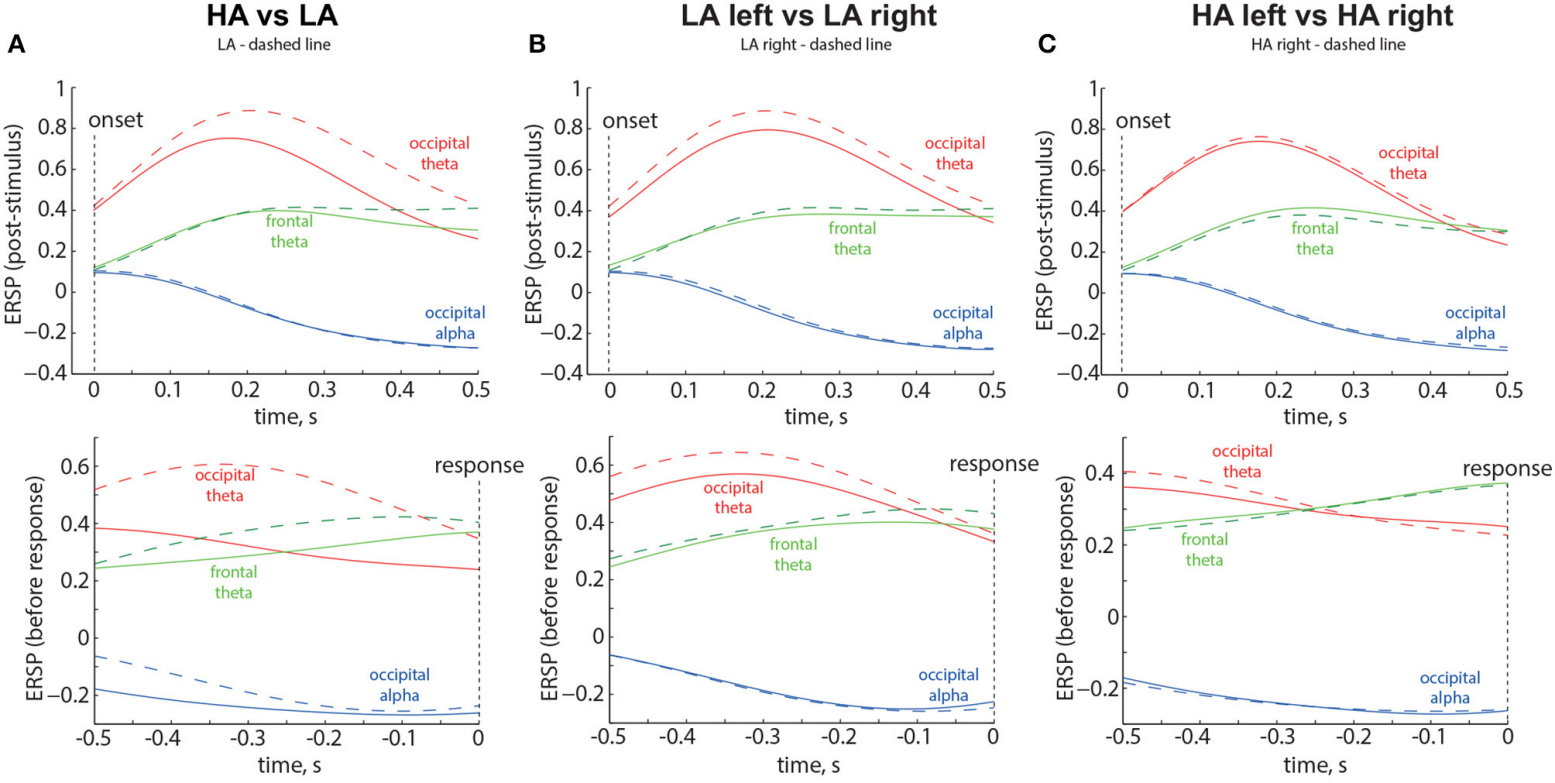
Time evolution of the ERSP in the Experiment 1 for LA and HA stimuli **(A)**, left- and right-oriented LA stimuli **(B)**, and left- and right-oriented HA stimuli **(C)**. The time evolution of the Event-Related Spectral Perturbation (ERSP) in three spatial-frequency areas of interest is depicted in the top panels, representing the intervals following the stimulus onset, and in the bottom panels, representing the intervals preceding button pressing. The results displayed are the grand average across 58 participants. Vertical dotted lines indicate the moments of the stimulus onset and response. Different colors correspond to the spatial-frequency areas of interest. The legend at the top of each panel provides the definition of the dashed line.

Based on the results of statistical analysis, we defined three spatial-frequency areas of interest. The *occipital alpha* reflected ERSP averaged across the occipital sensors (O1, Oz, O2, P7, P3, Pz, P4, P8) in the frequency band of 8 − 13 Hz. The *occipital theta* reflected ERSP averaged across the occipital sensors (P7, O1, Oz, O2, P8) in the frequency band of 1 − 7 Hz. The *frontal theta* reflected ERSP averaged across the frontal sensors (Fp1, Fp2, F3, Fz, F4, FC1, FC2) in the frequency band of 1 − 7 Hz. We illustrated the time evolution of the ERSP in three spatial-frequency areas of interest during the intervals following the stimulus onset and preceding button pressing. The results are shown in Figure 6 as the grand average across 58 participants. Vertical dotted lines indicate the moments of the stimulus onset and response. Different colors represent the spatial-frequency areas of interest.

#### 3.3.1. HA vs. LA stimuli1

The results are shown in the Figure 6A. The ERSP corresponding to the LA stimuli is shown by the dashed lines. Obtained results provide the following insights: First, *occipital theta* shows an increase for both ambiguities but exhibits higher values for LA stimuli (solid red line in Figure 6A). Second, *frontal theta* tends to be slightly higher for LA stimuli during the 0.3-s period before the decision (Figure 6A, bottom panel). Third, for HA stimuli, *frontal theta* tends to exceed *occipital theta* during the 0.35-s period before the decision (Figure 6A, bottom panel). Fourth, *occipital alpha* decreases for both ambiguities but shows lower values for HA stimuli during the 0.5-s period before the decision (dashed blue line in Figure 6A, bottom panel).

#### 3.3.2. Left-oriented vs. right-oriented LA stimuli

The results are shown in the Figure 6B. The ERSP corresponding to the right-oriented LA stimuli is shown by the dashed lines. Obtained results provide the following insights: First, *occipital theta* ERSP exhibits higher values for the right-oriented LA stimuli throughout the entire trial (red dashed line in Figure 6B). Second, *occipital theta* ERSP exceeds *frontal theta* ERSP throughout the entire trial. Third, no changes in the *frontal theta and occipital alpha* ERSP are observed between the left- and right-oriented LA stimuli.

#### 3.3.3. Left-oriented vs. right-oriented HA stimuli

The results are shown in the Figure 6C. The ERSP corresponding to the right-oriented HA stimuli is shown by the dashed lines. Obtained results provide the following insights: First, *frontal theta* ERSP starts to exceed *occipital theta* ERSP during the 0.3-s period before the decision (bottom panel in the Figure 6C). Second, no changes in the ERSP are observed between the left- and right-oriented HA stimuli.

**Figure 7:**
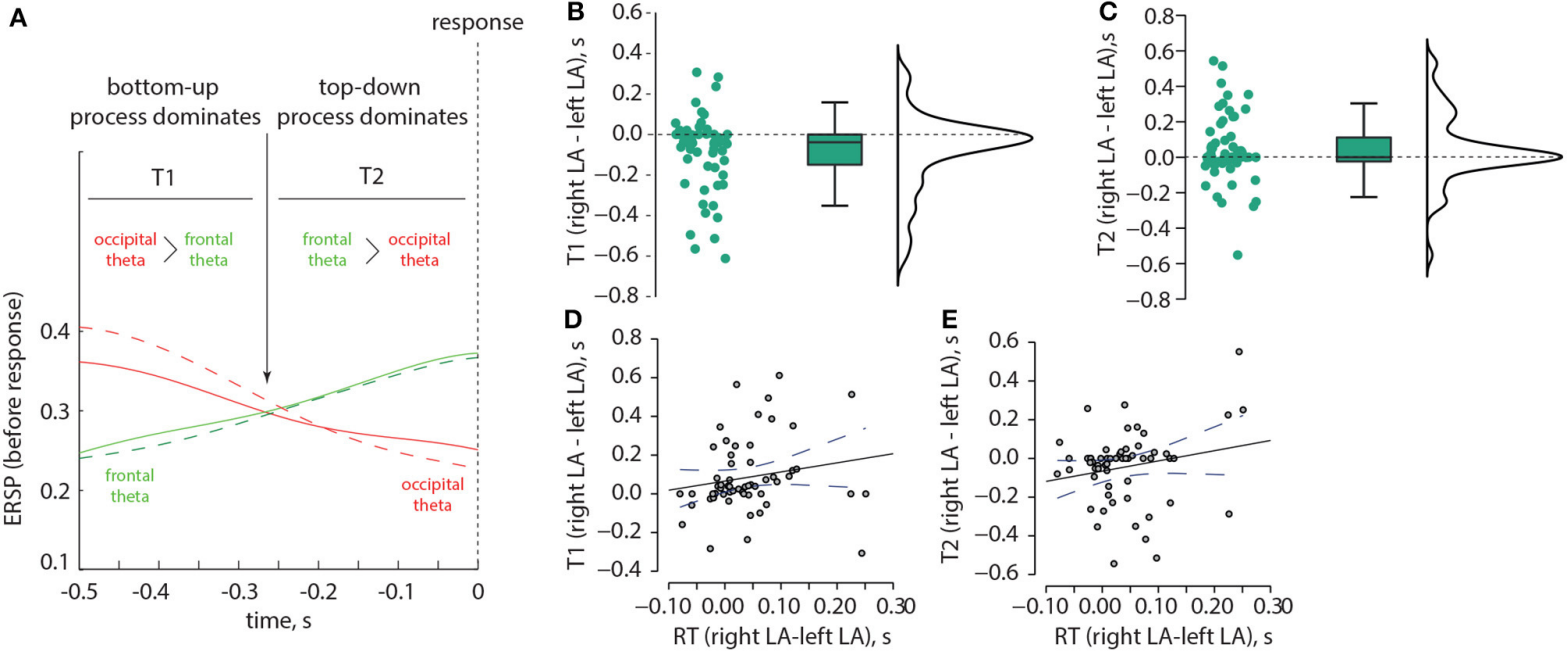
Correlation between the ERSP and RT in the Experiment 1. Schematic illustration of two time intervals, T1 and T2, which correspond to different ratios between the occipital and frontal ERSP in the theta-band **(A)**. The distributions of pairwise differences in T1 **(B)** and T2 **(C)**, respectively, are shown between the left-oriented and right-oriented LA stimuli. Panel **(D)** displays the correlation between the change in RT and the change in T1, while panel **(E)** depicts the correlation between the change in RT and the change in T2.

Based on these results, we examined two time intervals, T1 and T2 (Figure 7A). T1 corresponds to the situation when the occipital theta ERSP exceeds the frontal theta ERSP, while T2 corresponds to the situation when the frontal theta ERSP exceeds the occipital theta ERSP. We investigated whether T1 and T2 differed between the left-oriented and right-oriented stimuli. The non-parametric Wilcoxon test revealed a significant negative difference in T1 between the left-oriented and right-oriented stimuli: *z* = −3.384, *p* < 0.001 (Figure 7B). However, T2 did not show a significant difference between these two types of stimuli: *z* = 1.797, *p* = 0.073 (Figure 7C). Additionally, we used Spearman's rho correlation to assess the relationship between the difference in T1 and T2 and the change in RT. We found a positive correlation between the difference in T1 and the change in RT: ρ = 0.317, *p* = 0.015 (Figure 7D), indicating that individuals with longer T1 tended to have longer RTs, and vice versa. Conversely, the difference in T2 did not show a correlation with the change in RT: ρ = 0.167, *p* = 0.211 (Figure 7E).

### 3.4. Response time in the Experiment 2

In experiment 2, we analyzed the median RT using a repeated-measures ANOVA with three within-subject factors: ambiguity, orientation, and drawing. We observed significant main effects of ambiguity and orientation. There were significant interaction effects of drawing * orientation, and ambiguity * drawing * orientation. Effect of the other factors on RT was insignificant (Table 2).

**Table 2:** The main effects of the ambiguity, orientation, drawing, and their interaction on the median response time in the Experiment 2 (ANOVA summary).

**Factors**	***dF*_1_**	***dF*_2_**	**Mean square**	***F***	***p***
Orientation (left vs. right)	1	17	0.072	8.491	0.01^*^
Drawing (classical vs. mirrored)	1	17	< 0.001	0.062	0.807
Ambiguity * orientation	1	17	0.004	1.645	0.217
Ambiguity * drawing	1	17	0.001	0.489	0.494
Orientation * drawing	1	17	0.062	6.288	0.034^*^
Ambiguity* orientation * drawing	1	17	0.05	10.12	0.005^*^

^*^significant with *p* < 0.05.

**Figure 8:**
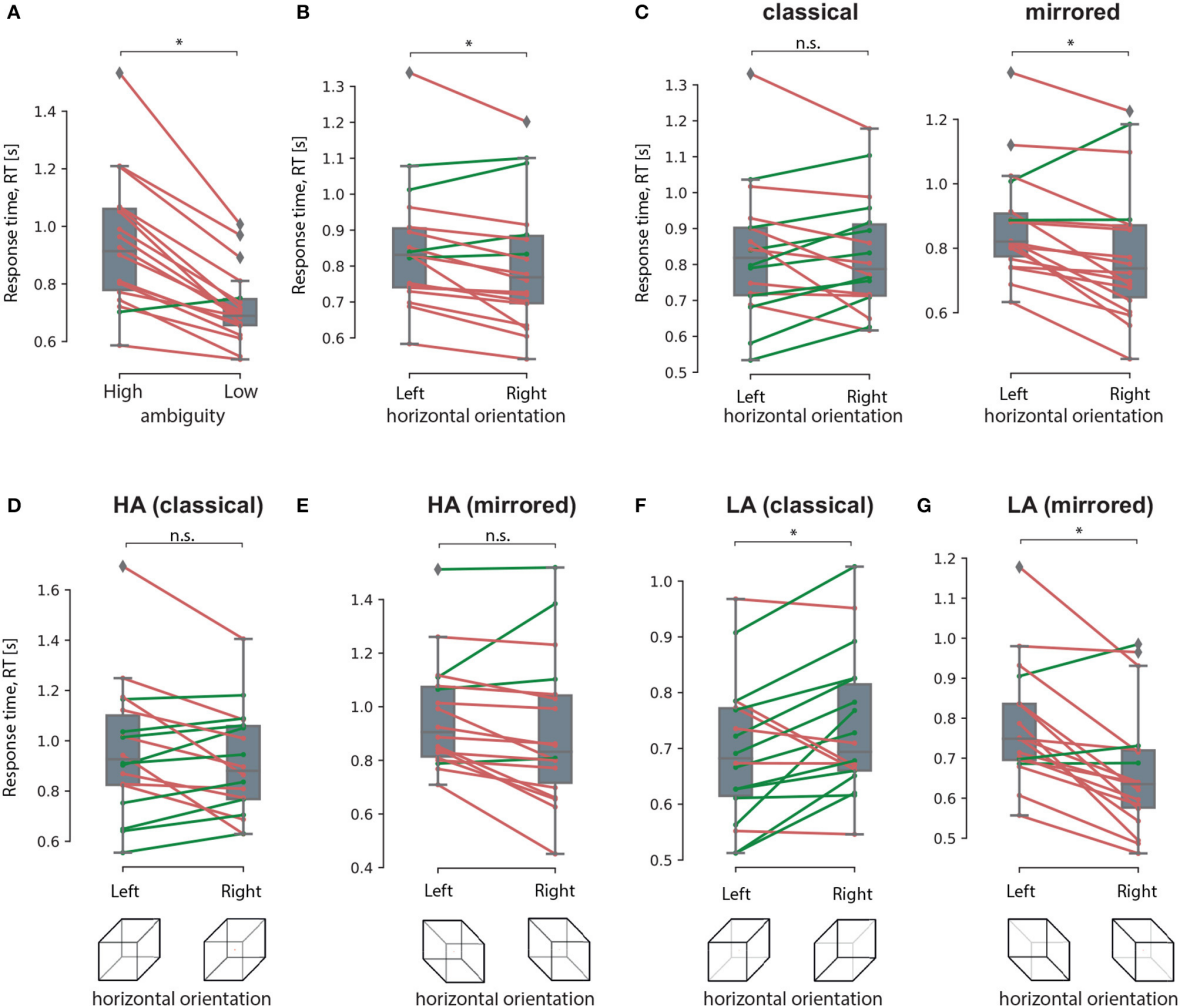
Response time analysis in The experiment 2. A result of the *post-hoc* comparisons of the median RT between HA and LA stimuli **(A)**, between the left-oriented and right-oriented stimuli **(B)**, between the left- and right-oriented stimuli in classical and mirrored drawing **(C)**, between the left-and right-oriented HA stimuli in classical **(D)** and mirrored **(E)** drawing, between the left- and right-oriented LA stimuli with classical **(F)** and mirrored **(G)** drawing. Group data is shown as a box-and-whiskers diagram illustrating median and 25–75 percentiles. Green and red lines illustrate individual RT change between the conditions. ^*^p < 0.05 (uncorrected) via a repeated measures ANOVA and the *post-ho*c *t*-test. n.s., not significant.

The *post-hoc* analysis revealed that RT for the HA stimuli (M = 0.93 s, SD = 0.22 s) was higher than the RT for the LA stimuli (M = 0.71 s, SD = 0.12 s): *t*(17) = 6.952, *p* < 0.001 (uncorrected) (Figure 8A). Studying the main effect of the orientation, we observed that subjects responded faster to the right-oriented stimuli (M = 0.8 s, SD = 0.18 s) than to the left-oriented stimuli (M = 0.85 s, SD = 0.17 s): *t*(17) = 2.941, *p* = 0.01 (uncorrected) (Figure 8B). Considering the interaction effect of orientation and drawing, we found that RT depended on the orientation in different ways depending on the drawing. For the classical drawing (similar to Experiment 1), RT did not differ between the left- and the right-oriented stimuli:*t*(17) = 0.142, *p* = 0.88 (uncorrected) (Figure 8C, left panel). For the mirrored drawing, RT for the left-oriented stimuli (M = 0.87 s, SD = 0.16 s) exceeded one for the right-oriented stimuli (M = 0.78 s, SD = 0.21 s): *t*(17) = 3.525, *p* = 0.003 (uncorrected) (Figure 8C, right panel). Finally, the significant interaction effect of all factors evidenced that RT changed between the left- and right-oriented stimuli depending on their ambiguity and drawing. The *post-hoc* analysis revealed that RT for HA-stimuli did not differ between the orientations for the both drawings (Figures 8D, E). For the LA stimuli, RT differed between the orientations in the different way depending on the drawing. For the classical drawing, subjects responded faster to the left-oriented LA stimuli than to the right-oriented ones (Figure 8F). For the mirrored drawing, they responded faster to the right-oriented LA stimuli than to the left-oriented ones (Figure 8G).

## 4. Discussion

We conducted a perceptual decision-making task using ambiguous visual stimuli, specifically Necker cubes (Experiment 1). Participants were instructed to determine the orientation of the stimulus (left or right) and indicate their decision using their left or right hand. It is important to note that we excluded stimuli that were completely ambiguous from our study. As a result, the overall accuracy rate exceeded 90%, indicating a reliance on sensory information for decision-making and confirming the goal-directed nature of the task. The main finding of this experiment is that for stimuli with low ambiguity, participants responded faster to the left-oriented stimuli. However, for stimuli with high ambiguity, the response time remained relatively consistent between the left- and right-oriented stimuli.

In the classical drawing of the Necker cube used in Experiment 1, the left-oriented stimulus has a from-above-perspective (FA), while the right-oriented stimulus has a from-below-perspective (FB). Therefore, both the orientation and perspective may contribute to the response time (RT) bias. To address this uncertainty, we conducted Experiment 2, where we presented both FA and FB projections for both left- and right-oriented stimuli. For the stimuli with low ambiguity, we found that participants had shorter response times for the FA perspective (both left- and right-oriented stimuli) compared to the FB perspective. Consistent with the findings of Experiment 1, this effect diminished for stimuli with high ambiguity. Together, our behavioral results demonstrate a perceptual bias toward the from-above (FA) perspective in goal-directed Necker cube viewing. This bias remains present for stimuli with low ambiguity but diminishes as ambiguity increases.

Obtained behavioral results suggest that the brain utilizes different processing strategies for stimuli with low and high ambiguity. Specifically, in the case of low ambiguity, the processing strategy is more sensitive to the perspective compared to high ambiguity. To gain further insights into these processing strategies, we conducted a comparison of brain activity between low ambiguity (LA) and high ambiguity (HA) processing. Our findings reveal several important insights. First, processing the Necker cube, regardless of ambiguity level, leads to a reduction in alpha-band power across the occipital sensors (alpha-band event-related desynchronization, ERD). Additionally, ambiguous stimuli elicit higher ERD amplitudes compared to unambiguous stimuli (Figure 3C). Second, processing the Necker cube results in increased low-frequency (delta and theta) spectral power across the occipital sensors (low-frequency-band event-related synchronization, ERS). Unambiguous stimuli exhibit higher ERS amplitudes compared to ambiguous stimuli (Figures 3A, B). Third, processing ambiguous stimuli is associated with higher low-frequency band power in the frontal-midline electrodes, while processing unambiguous stimuli involves higher low-frequency band power in bilateral occipital sites (Figure 3B). Fourth, when processing low-ambiguity (LA) stimuli, the occipital EEG power in the low-frequency band increases for right-oriented stimuli compared to left-oriented stimuli (Figure 5). Fifth, there is no difference in neural activity between processing HA stimuli with left and right orientation. Finally, we observed that theta-band power at frontal electrodes begins to exceed the power at occipital electrodes before the decision-making process. The duration for which theta-band power at occipital electrodes dominates differs between left-oriented and right-oriented LA stimuli and explains the difference in response time observed between them (Figure 7).

The higher alpha-band ERD may reflect various processes, including alertness elicited by the stimulus and retrieval of information from memory for encoding (Klimesch et al., 2011). The low-frequency occipital ERS may reflect attentional processing of the external stimulus and the accumulation of sensory evidence in favor of a particular interpretation (van Vugt et al., 2012). Based on these results, we suggest that interpreting ambiguous stimuli engages more resources, as indicated by the higher alpha-band ERD at occipital electrodes, but relies on internal processing, as reflected by theta-band power peaks at the frontal midline electrodes. Unambiguous stimuli require greater attention to external information and necessitate the accumulation of more evidence to encode an object in the visual area, as evidenced by the high occipital theta-band ERS. According to the predictive coding theory, the brain compares the representation of the stimulus in the sensory areas with the template stored in working memory. If they match, the observer makes a decision; otherwise, they continue gathering sensory evidence. Therefore, the higher amplitude of the occipital ERS may reflect the need for more information to encode the right-oriented cube with a from-below (FB) perspective, as its template is less common for the observer compared to the template for the left-oriented cube with a from-above (FA) perspective. This is further supported by the fact that occipital theta-band power dominates for a longer time when processing stimuli with the from-below perspective. This suggests that sensory processing and evidence accumulation take longer for these stimuli, resulting in increased response time.

Our findings coincides with the previous findings reported by Kornmeier et al. (2017b). In their study, which focused on totally ambiguous Necker cubes, they also observed a perceptual bias in favor of the from-above (FA) perspective (Kornmeier et al., 2017b). However, it is important to note that their study primarily examined involuntary switching between interpretations rather than goal-directed viewing and included only ambiguous images. Therefore, the authors concluded that the a priori bias in favor of the FA perspective is a result of top-down processing. This process assists in perceiving the Necker cube stimulus as a three-dimensional object by utilizing templates stored in working memory. Additionally, the bias may reflect a common statistical occurrence: we tend to look downward more frequently than upward at objects. The absence of the FA bias in patients with autism spectrum disorder supports this idea, as these individuals struggle with integrating spatial context (Allen and Chambers, 2011) and prior perceptual experiences (Mitchell and Ropar, 2004). Consequently, their perception relies more on bottom-up components rather than top-down processes.

Thus, our results provide insights into the distinctions between goal-oriented processing (our study) and involuntary processing (Kornmeier et al.) of ambiguous information. According to the predictive coding theory, the brain interprets information by continuously comparing sensory evidence with templates stored in working memory (Kok and de Lange, 2015). Sensory information is acquired until a match is found with the perceptual template. In involuntary processing, sensory evidence does not significantly differ between the interpretations, rendering both templates equally viable. Consequently, processing becomes predominantly driven by internal factors. Given our previous experiences, the template associated with the from-above (FA) perspective is more likely to be applied. During goal-directed processing, sensory information carries distinguishing features between different interpretations. In goal-directed behavior, the observer controls information processing to ensure the alignment between the presented information and internal template. Participants correctly identify the Necker cube's interpretation for both FA and FB perspectives, indicating that the templates are selected appropriately based on the sensory information. The increased response time for stimuli with the less common from-below (FB) perspective reflects the need for acquiring more information to achieve this alignment. Thus, in goal-directed behavior, the longer response time observed for the less common from-below (FB) perspective compensates for the a priori top-down perceptual bias in favor of the more usual from-above (FA) perspective that dominates during involuntary perception.

Our study has several potential limitations. First, the sample size in Experiment 2 is small, which may introduce between-subject variability and affect the results of statistical analysis. To address this limitation, we sampled participants from a population with a narrow age range and ensured a 50/50 gender balance. However, future studies with a larger participant group would enhance the reliability of our findings. Second, in Experiment 2, we did not record EEG signals. Therefore, we could not compare neural activity between FA and FB perspectives. Conducting further studies with a larger group of participants and including EEG recordings would allow for a more comprehensive examination of the neural correlates associated with different perspectives. Additionally, future studies should consider incorporating psychological tests to estimate the bias between top-down and bottom-up processing in each participant. If our hypothesis holds true, participants with a high bias toward the bottom-up component would likely demonstrate a lower RT bias, and vice versa. This would provide a deeper understanding of individual differences in processing strategies. Finally, we suggest including eye-tracking in future experiments. According to the literature, pupil size and saccades can distinguish between different processing strategies, such as procedural and insight-based processing (Salvi et al., 2020). Therefore, incorporating eye-tracking measures may offer further behavioral evidence to support our hypothesis.

Finally, we discuss the potential role of emotions in perceptual decision-making tasks involving ambiguous Necker cube stimuli. Growing evidence suggests that even simple perceptual decisions are influenced by emotions. The emotional effect arises from two different sources: the emotional state of the decision-maker and the emotional content of a stimulus (Mériau et al., 2006). The effect of emotions is studied in preferential and ethical decisions, which are believed to involve subjectivity and are hence more sensitive to emotions. In the perceptual decisions that are based on the objective sensory evidence (Heekeren et al., 2008), emotional aspects are less studied.

The Necker cube stimulus is an example of a perceptual decision-making task. Unlike other perceptual decision-making paradigms, Necker cube images are emotionally neutral. What is more important, different interpretations (left- and right-oriented projections) have almost the same morphology. So, even if the Necker cube has an emotional impact on the observer, the emotional impact of different orientations barely differs, unlike the Rubin vase or face-non-face paradigm. Therefore, we expect that the state of the person will be the main source of emotional impact, rather than the emotional content of a stimulus.

Our hypothesis is that the emotional state of an observer may change their problem-solving strategy for perceptual decisions when identifying interpretations of ambiguous stimuli. There is a view that people solve problems through either an analytical strategy or insight (Fleck and Weisberg, 2013). An analytical strategy means that solving progresses gradually, moving step by step toward a solution (Laukkonen and Tangen, 2017). Insight is the sudden solution to a tough problem, a sudden recognition of a new idea, or a sudden understanding of a complex situation. There is a belief that solutions found through insight are often more accurate than those found through step-by-step analysis (Salvi et al., 2016). According to Gestalt theorists, the insight problem-solving experience is similar to the perceptual switch experienced when reinterpreting ambiguous figures (Salvi et al., 2020). When looking at ambiguous figures, observers encounter perceptual rivalry, switching between different possible perceptual alternatives. A recent study demonstrated that participants who better identified two alternative perspectives in ambiguous images were also better at solving insight problems. Moreover, insight problem-solving ability improved when participants experienced the ambiguous drawing of the Necker cube (Laukkonen and Tangen, 2017). Thus, insight problem-solving and perceptual rivalry may rely on similar cognitive processes. Regarding insight problem-solving, some studies show its association with many types of emotions (Shen et al., 2016). In particular, negative and positive emotions may facilitate insight problems in some circumstances (Li et al., 2013, 2020).

In summary, we expect similarities in the effects of emotions on Necker cube processing and their effects on insight problem-solving which are reported in literature. Future studies will test this hypothesis by estimating the emotional state of individuals during the perceptual decision-making Necker cube task and relating it to their performance at the behavioral level.

## 5. Conclusion

Considering that the brain interprets information by continuously comparing sensory evidence with templates stored in working memory, we can highlight the following distinction between goal-oriented and involuntary processing of ambiguous Necker cube stimuli.

During involuntary processing, sensory evidence does not significantly differ between the interpretations, making both templates equally plausible. Consequently, processing becomes primarily driven by internal factors. Based on our previous experiences, the template associated with the more common from-above (FA) perspective is more likely to be applied.

In goal-directed processing, sensory information contains distinguishing features that differentiate between different interpretations. The observer actively controls information processing to ensure alignment between the presented information and the internal template. Participants successfully identify the Necker cube's interpretation for both from-above (FA) and from-below (FB) perspectives, indicating appropriate template selection based on sensory information. However, the increased response time observed for stimuli with the less common from-below (FB) perspective suggests the need for gathering additional information to achieve this alignment.

Therefore, in goal-directed behavior, the longer response time observed for the less common from-below (FB) perspective compensates for the inherent top-down perceptual bias favoring the more frequent from-above (FA) perspective, which dominates during involuntary perception.

## Data availability statement

The data that support the findings of this study are available from the corresponding author, VM, upon request.

## Ethics statement

The studies involving humans were approved by Local Ethics Committee of the Lobachevsky State University of Nizhny Novgorod (ethical approval number 2, dated 19 March 2021). The studies were conducted in accordance with the local legislation and institutional requirements. The participants provided their written informed consent to participate in this study. Written informed consent was obtained from the individual(s) for the publication of any potentially identifiable images or data included in this article.

## Author contributions

VM and AH designed the study and wrote manuscript. AK analyzed data. SG and VK supervised experiments. AS, NG, AB, and VG conducted experiments. All authors contributed to the article and approved the submitted version.
